# Flock-level risk factors for outbreaks of infectious arthritis in lambs, Norway 2018

**DOI:** 10.1186/s13028-020-00561-z

**Published:** 2020-11-23

**Authors:** Marit Smistad, Cecilia Wolff, Tore Tollersrud, Vibeke Tømmerberg, Clare Phythian, Annette Hegermann Kampen, Hannah Joan Jørgensen

**Affiliations:** 1 Norwegian Veterinary Institute, Ullevålsveien 68, 0454 Oslo, Norway; 2 TINE SA, Postboks 7, 0901 Oslo, Norway; 3 Norwegian Meat and Poultry Research Centre, Lørenveien 38, 0513 Oslo, Norway; 4 Norwegian University of Life Sciences, Faculty of Veterinary Medicine, Institute for Production Animal Clinical Science, Svebastadveien 112, 4325 Sandnes, Norway

**Keywords:** Arthritis, Joint ill, Management, Ovine, Questionnaire, SDSD, *Streptococcus dysgalactiae* subspecies *dysgalactiae*

## Abstract

**Background:**

Outbreaks of infectious arthritis in young lambs associated with *Streptococcus dysgalactiae* subspecies *dysgalactiae* (SDSD) lead to reduced animal welfare, increased use of antibiotics and economic losses for sheep farmers. Understanding risk factors is essential when developing strategies to prevent such outbreaks. This questionnaire-based cross-sectional study classified sheep flocks of respondents as cases or controls. Flock-level risk factors for outbreaks of infectious arthritis were assessed using a multivariable logistic regression model.

**Results:**

Eighty-four of 1498 respondents (5.6%) experienced an outbreak of infectious arthritis in their flock in 2018, the year of study. Factors associated with a higher risk of outbreak were larger flock size (OR 1.3, 95% CI 1.1–1.4, per 100 lambs), plastic mesh flooring in the lambing pen (OR 3.0, 95% CI 1.7–5.3) and a lambing percentage greater than 200 (OR 2.0, 95% CI 1.1–3.5). Flocks where farmers observed infections around the ear tags of lambs also had an increased risk of outbreak (OR 2.6, 95% CI 1.6–4.3).

**Conclusions:**

The risk factors identified in this study are characteristic of modern and intensively managed sheep farms in Norway. A distinguishing feature of Norwegian sheep farming is winter housing and indoor lambing. One might expect that this in itself is a risk factor because of high stocking densities during lambing. However, outbreaks of infectious arthritis in young lambs are reported by the industry to be a more recent phenomenon. The current study indicates that intensification of indoor management systems with larger flocks and higher production per ewe may predispose to outbreaks. The results provide a basis for further studies on transmission dynamics of SDSD in sheep flocks with indoor lambing.

## Background

Over the past 10 years, outbreaks of infectious arthritis (joint ill) in young lambs have been a growing concern for the Norwegian sheep industry. In some farms, up to 40% of the lambs have been affected shortly after birth [1]. Not only does this present a serious animal welfare issue, but the scale and nature of antibiotic use in affected flocks is contrary to the Norwegian policies on antimicrobial use for livestock [2].

Although joint-swelling and lameness of non-infectious origin may occur in sheep, a sudden onset and rapid within-flock spread of disease in young lambs, is characteristic of infectious arthritis [3]. The disease can have several bacterial causes with *Erysipelothrix rhusiopathiae*, *Staphylococcus aureus* and *Streptococcus dysgalactiae* subspecies *dysgalactiae* (SDSD) as the major species. Some authors consider *E. rhusiopathiae* the most common cause of arthritis in lambs [4]. This infection is occasionally seen in Norway, also in lambs below 1 month of age [5], but it typically presents in lambs between 2 and 6-months of age [3, 4]. *S. aureus* is also a relatively common cause of arthritis in lambs, but outbreaks are often a complication of tick-associated pyemia in lambs on pasture [4].

SDSD has been identified as the most important cause of outbreaks of infectious arthritis in young lambs in England and Wales [3, 6], and as a cause of outbreaks of polyarthritis in lambs in Australia [7], New Zealand [8] and Spain [9]. Typically, SDSD arthritis affects lambs under 4 weeks of age [10], and clinical features include acute lameness, fever and recumbency. The lambs are often dull and unthrifty, and some show signs of secondary pneumonia or meningitis [3, 10].

In order to document the microbiological causes of outbreaks of infectious arthritis in young lambs in Norway, the Norwegian Sheep Health Service and veterinary practitioners conducted a pilot study during the lambing seasons of 2016, 2017 and 2018. They visited 19 flocks, 12 of which experienced an outbreak at least one of the seasons. Approximately two thirds of the clinical cases occurred within the first week of life [1]. Upon bacteriological culturing, SDSD was identified from joint aspirates in 11 of the 12 flocks (Tømmerberg et al., unpublished data), indicating that SDSD is the main causative agent of outbreaks of infectious arthritis in young lambs in Norway.

Previous studies on SDSD have suggested unhygienic conditions in the lambing pen [3, 11], high stocking densities [10] and early ear tagging to be associated with an increased risk of outbreaks of infectious arthritis in lambs [12, 13]. Contaminated stomach tubes [14] and inadequate hygiene when providing lambing assistance [15] have been proposed as possible routes of bacterial transmission between animals.

In Norway, anecdotal reports from the sheep industry suggest that outbreaks of SDSD joint ill are mainly associated with large, intensively managed sheep flocks with many hundreds of lambs born indoors within a few weeks [1]. Winter-housed sheep are fed high quality silage and concentrate, and a lambing percentage of 250 is common in these flocks. Stocking densities are high, and the indoor environment can be unfavourable at the peak of the lambing season. However, well managed flocks with good hygiene have also experienced outbreaks [1].

The Norwegian sheep industry wishes to develop evidence-based management strategies to prevent outbreaks of infectious arthritis in sheep flocks with indoor laming. The objective of the study, therefore, was to perform a survey to identify flock-level risk factors for outbreaks of infectious arthritis in lambs under Norwegian management conditions.

## Methods

### Structure of the Norwegian sheep industry

In 2018, there were approximately 14,000 sheep farms in Norway [16], and 40% of these were members of the Norwegian Sheep Recording System (NSRS) [17]. The average flock size among NSRS-members was 86 winter-housed sheep, and 6% of the flocks have more than 200 ewes [17]. The animals are kept for meat and wool, and the main breed is Norwegian White Sheep, a composite crossbreed accounting for about 70% of the national population. Typically, flocks are housed during the winter season from mating until 1–2 weeks after lambing. Lambing starts between March and May depending on the local climate. The sheep and their lambs are let out onto spring pasture for a few weeks, before they are sent to summer pasture in outfield grazing areas in the woods or mountains or kept on lowland grazing areas. The lambs are slaughtered in the period between August and November, and the average carcass weight is 18.4 kg [18]. In Norway, it is mandatory to ear-tag lambs within 30 days after birth, and most sheep farmers tag their lambs the first week of life. Tail docking and castration is prohibited according to the animal welfare legislation [19].

### Study design and data collection

This cross-sectional study of the lambing season of 2018 was based on a survey. On the 14th of March 2019, the Norwegian Sheep Health Services distributed the online questionnaire to all members of the NSRS with a registered e-mail address (n = 5374). The questionnaire was also made available by link on the web page of the Norwegian Meat and Poultry Research Centre (www.Animalia.no). The survey closed on the 25th May 2019, after one email reminder.

### Questionnaire

The questionnaire, created in Questback (www.Questback.com), included 86 questions (Additional file 1), and took about 20 min to complete. Most questions were closed, or semi-closed, and where relevant, space was provided for comments. Before distribution, three sheep farmers, with no history of infectious arthritis outbreaks in their flocks, tested the questionnaire to ensure clarity.

To provide data on suspected risk factors, the 86 questions covered: (i) flock characteristics (ii) housing (iii) feeding routines (iv) management practices in general and during lambing for the season of 2018, and (v) the occurrence of infectious arthritis during the previous four lambing seasons (2015–2018). Data were collected and stored according to the General Data Protection Regulation (GDPR) [20]. Farmers (respondents) could choose to answer the questionnaire anonymously by providing their postal code instead of their farmer-id.

### Definition of case and control flocks

Survey data from flocks with more than 40 lambs born during the lambing season of 2018 were reviewed for inclusion as cases or controls. The following inclusion criteria for case-flocks were used: (i) the farmer reported that five percent or more of the lambs were affected with arthritis in the lambing season of 2018, (ii) the affected lambs were 4 weeks or younger and (iii) the clinical signs of affected lambs were lameness in combination with either joint swelling, pyrexia, dullness or respiratory signs. Farmers that reported more than five percent of lambs affected with arthritis but described lameness and interdigital swelling as the only clinical signs, were excluded from the analyses because those symptoms are more likely to be associated with interdigital abscesses than arthritis. Survey data from remaining respondents were included as control-flocks.

### Data management

Raw data were exported and stored in Excel (Microsoft Corp, Redmond, WA, USA, 2016) and analysed with Stata (Release 14.2, Stata Corp LLC, USA, 2015). Variable categories with five or fewer observations were amalgamated when biologically or logically possible, or not included the multivariable model. When feasible, multiple questions within the same topic were combined into one variable, e.g. the variable “environment in the shed” was created from four statements in the questionnaire regarding the environment.

For flocks with missing data on flock size, lamb mortality and breed, the information was electronically retrieved from the NSRS, when available. Variables with more than 10% missing observations were not considered for multivariable analysis. Respondents with more than 15% missing variables were excluded from the analyses.

### Data analysis

Before analysis, the hypothetical relationship between the outcome and exposures was outlined in a causal diagram, based on literature review, biological knowledge and clinical observations of the research team. Categorical variables were described by cross tabulation against the outcome. Continuous variables were plotted against the outcome variable using boxplots. Linearity was assessed by plotting continuous variables against the logit transformed outcome with Lowess smoothing plots [21].

First, unconditional associations between the dependent variable and each of the potential risk factors were screened using Chi^2^-tests (categorical variables). Flock size was rescaled by dividing it by 100 to aid interpretation of the OR. Variables with a P-value ≤ 0.2 were tested in the multivariable analysis. Spearman rank correlations (categorical ordinal), tabulation (categorical nominal) and Pearson correlations (continuous) were used to assess collinearity between the predictors [21]. If two variables showed collinearity (r > 0.7, where applicable) the one with the lowest P-value or suspected highest biological relevance was kept for further analysis.

A multivariable logistic regression model was used to evaluate the risk factors for being a case flock. The model was built using manual backwards elimination, with the logit function. Variables were removed from the model based on likelihood ratio-test at each step, with P < 0.05 as a criterion for retention [21].

To assess confounding, variables excluded during the reduction were re-entered one at a time when all remaining variables were significant. A variable was considered a confounder if there was a greater than 20% change in any coefficients’ estimates when the variable was included. Biologically plausible interaction terms between main effects were tested in the model. The fit of the model was evaluated with Hosmer–Lemeshow goodness of fit test with the data partitioned into 10 deciles. Outliers and influential observations were identified by examinations of observations with Pearson residuals > 2 or < − 2, deviance residuals > 2 or < − 2 or with leverage (hat) > 3 * mean hat.

The representativeness of the sheep flocks of respondents was examined by comparing the breed composition, the mean flock size and the mean lamb mortality with averages reported by the NSRS [17]. The geographical distribution of respondent flocks per county was visually compared with the distribution of all sheep flocks in Norway. Flock size and lamb mortality percentage of the flocks in the data set used in the multivariable analysis were compared to the complete dataset including all respondents to assess differences that could potentially bias the results.

## Results

### Study population

A total of 1761 farmers responded to the questionnaire. Of these, 1490 responded to the e-mail, giving a response rate of 27%. In addition, 271 farmers responded via the link on the web page. Data from respondents were excluded from the analyses if the flock had less than 40 ear-tagged lambs (n = 210), answers had more than 15% missing values (n = 33), were duplicates (n = 12), or the farmer reported that more than five percent of the lambs were affected but described lameness and interdigital swelling as the only clinical signs indicating a problem of interdigital abscesses rather than arthritis (n = 8). Among the 1498 flocks that met the inclusion criteria and were available for descriptive statistics, 84 (5.6%) were classified as case flocks. The final dataset, without missing values, used for the multivariable analyses included 77 case flocks and 1178 control flocks.

### Flock characteristics and management practices

The number of ear-tagged lambs was used as an indicator of flock size. The flock size ranged from 40 to 1323 ear-tagged lambs. The median flock size was 226 ear-tagged lambs [interquartile range (iqr) 133–371] in case flocks and 134 (iqr 84–226) in control flocks. There was an association between being a case flock and larger flock size in the univariable analysis (P < 0.001). The case flocks had a median overall lamb mortality of 2.5% (iqr 1.6–4.3) while the control flocks had a median overall lamb mortality of 1.9% (iqr 0.9–3.3).

Thirty-three of the 44 explanatory variables tested in the univariable analysis are presented in Tables 1, 2, 3, 4, and 5. Any association between outbreaks and the presence of other animal species on the farm (10 variables) were tested in the univariable analysis, but not in the multivariable model due to more than 15% missing values. A question about the total indoor area in the shed during the winter season was part of the questionnaire (not shown), but could not be used because many respondents commented that they use additional areas during lambing or had difficulty in defining the relevant areas. Many also left the question blank.

**Table 1:** Description of flock data variables tested in univariable screening (Chi^2^-test)

Variable	Categories	Total		Case flocks (n = 84)		Control flocks (n = 1414)		
		n	%	n	%	n	%	P-value
Number of lambs (ear-tagged, categorized)^a^	< 200	1014	67.7	36	42.9	978	69.2	< 0.0001
	200–500	427	28.5	36	42.9	391	27.7	
	> 500	57	3.8	12	14.3	45	3.2	
Outbreak of infectious arthritis before 2018^c^	No	1286	85.9	18	21.4	1268	89.7	< 0.0001
	Yes	212	14.2	66	78.6	146	10.3	
Lambing percentage^b^	≤ 200	592	39.7	19	22.6	573	39.7	0.001
	> 200	898	60.3	65	77.4	833	60.3	
Breed^b^	Breed other than Norwegian White Sheep	301	20.5	7	8.9	294	21.1	0.009
	Norwegian White Sheep	1171	79.6	72	91.1	1099	78.9	
Start of lambing season^b^	April	1181	79.2	62	73.8	1119	73.8	0.038
	May	189	12.7	9	10.7	180	12.7	
	March	121	8.1	13	15.5	108	7.7	
Length of lambing season^b^	< 4 weeks	921	61.5	44	52.4	877	62.1	0.076
	> 4 weeks	576	38.5	40	47.6	536	37.9	

^a^Not tested in the multivariable model due to collinearity with number of ear-tagged lambs (continuous, not shown)

^b^Tested in multivariable model

^c^Intervening variable, not tested in the multivariable model

**Table 2:** Description of variables for housing conditions tested in the univariable screening (Chi^2^-test)

Variable	Categories	Total		Case flocks (n = 84)		Control flocks (n = 1414)		
		n	%	n	%	n	%	P-value
Flooring type in lambing pen^a^	Metal mesh flooring	649	43.4	26	31.0	623	44.1	< 0.0001
	Plastic mesh flooring	314	21.0	41	48.8	273	19.3	
	Other/combinations	533	35.6	17	20.2	516	36.6	
Flooring type for lambs before let out onto pasture^b^	Metal mesh flooring	348	23.2	21	25.3	327	23.2	< 0.0001
	Plastic mesh flooring	145	9.7	26	31.3	119	8.4	
	Straw bed/deep litter	310	20.8	10	12.1	300	21.3	
	Other/combinations	691	46.3	26	31.3	665	47.1	
Bedding material in single pens^c^	Not using bedding material	355	23.4	20	24.1	335	23.8	0.005
	Straw	493	33.1	42	50.6	451	32.0	
	Sawdust	178	12.0	9	10.8	169	12.0	
	Hay	167	11.2	6	7.2	161	11.4	
	Other bedding materials or combinations	271	18.2	6	7.2	265	18.8	
	Not using single pens	27	1.8	0	0	27	1.9	
Age of the shed^a^	> 10 years	952	63.9	41	48.8	911	64.8	0.01
	Rebuilt/modernized the last 10 years	282	18.9	21	25.0	261	18.6	
	< 10 years	256	17.2	22	26.2	234	16.6	
Environment in the shed after lambing vs. before^a^	Dry	611	42.4	26	31.0	585	43.1	0.021
	More humid	426	29.6	24	28.6	402	29.7	
	More humid and dirtier	403	28.0	34	40.5	369	27.2	
Time spent in single pens after lambing^c^	≥ 3 days	840	56.2	38	45.2	802	56.8	0.031
	1–2 days	628	42.0	46	54.8	582	41.3	
	Not using single pens	27	1.8	0	0.0	27	1.9	
Group size (ewes before lambing)^a^	≤ 15	767	53.0	36	42.9	731	53.7	0.086
	16–30	441	30.5	28	33.3	413	30.3	
	> 30	238	16.5	20	23.8	218	16.0	
Housing type	Uninsulated	405	27.3	19	22.6	386	27.6	0.512
	Insulated	745	50.2	47	56.0	698	49.9	
	Other housing type, outdoors combination	333	22.5	18	21.4	315	22.5	

^a^Tested in multivariable model

^b^Intervening variable, not tested in the multivariable model

^c^Not tested in the multivariable model due to categories with five or fewer observations

**Table 3:** Description of variables for management at lambing tested in the univariable screening (Chi^2^-test)

Variable	Categories	Total		Case flocks (n = 84)		Control flocks (n = 1414)		
		n	%	n	%	n	%	P-value
Observed infections around ear tag^a^	No (never/rarely)	1103	73.7	44	52.4	1059	75.0	< 0.0001
	Yes (sometimes, often)	393	26.3	40	47.6	353	25.0	
Routines for colostrum supply^b^	Observe that they suck, not using stomach tubes	504	33.7	11	13.1	493	34.9	< 0.0001
	Observe that they suck, use stomach tubes routinely/when needed	931	62.2	67	79.8	864	61.1	
	Not consistent, no clear routines	62	4.1	6	7.1	56	4.0	
How often are stomach tubes used for colostrum supply^a, b^	Not using stomach tubes	550	36.8	15	17.9	535	37.9	< 0.0001
	Sometimes (1–10% of the lambs)	776	51.9	49	58.3	727	51.5	
	Relatively often (> 10% of the lambs)	170	11.4	20	23.8	150	10.6	
Disinfection of navels^a^	Never/rarely, sometimes	633	42.3	22	26.2	611	43.2	0.002
	Yes	864	57.7	62	73.8	602	56.8	
Age at ear tagging^a^	1 day	746	49.9	47	56.0	699	49.5	0.011
	2 days	370	24.8	27	32.1	343	24.3	
	≥ 3 days	379	25.4	10	11.9	369	26.2	
Statement: as far as possible the ewe and her lambs are left in peace during and immediately after lambing^a^	Fully agree	954	64.1	43	51.1	911	64.8	0.017
	Partly agree/disagree	535	35.9	40	48.2	495	35.2	
% of ewes needing assistance during lambing^a^	0–10%	443	30.2	16	19.3	427	30.9	0.031
	11–20%	430	29.3	23	27.7	407	29.4	
	> 20%	594	40.5	44	53.0	550	39.7	
Use of disinfectant on ear tag	No/sometimes	908	61.1	54	65.9	854	60.8	0.36
	Yes	578	38.9	28	34.2	550	39.2	

^a^Tested in multivariable model

^b^Not tested in the multivariable model due to collinearity with another variable (with the same letter)

**Table 4:** Description of variables for hygienic measures tested in the univariable screening (Chi^2^-test)

Variable	Categories	Total		Case flocks (n = 84)		Control flocks (n = 1414)		
		n	%	n	%	n	%	P-value
Hand hygiene: statement: “I always wash my hands and/or change gloves after handling diseased animals”^a^	Fully agree	1309	87.4	66	78.6	1243	88.0	0.012
	Partly agree/disagree	188	12.6	18	21.4	170	12.0	
Hand hygiene when performing lambing assistance^a^	Adequate (always hand wash or change of gloves)	1423	95.3	76	90.5	1347	95.6	0.03
	Inadequate (not consistent hand hygiene measures)	70	4.7	8	9.5	62	4.4	
Is the bedding material in the single pens changed between lambings?^b^	Usually/always	747	66.2	47	77.1	700	65.6	0.126
	Sometimes/never	354	31.4	14	23.0	340	31.9	
	Do not use single pens	27	2.4	0	0.0	27	2.53	
Are the single pens cleaned between lambings?	Always	99	29.3	7	35.0	92	28.9	0.563
	Sometimes/never	239	70.7	13	65.0	226	71.1	
How often are the bottles/stomach tubes cleaned?	Between every lamb	722	48.4	39	46.4	683	48.5	0.710
	Once daily	492	33.0	31	36.9	461	32.7	
	When needed	279	18.7	14	16.7	265	18.8	
How often is the shed cleaned (washed)?	Annually	988	66.0	57	67.9	931	65.9	0.710
	Less often than annually	509	34.0	27	32.1	482	34.1	

^a^Tested in multivariable model

^b^Not tested in the multivariable model due to categories with five or fewer observations

**Table 5:** Description of variables related to feeding tested in the univariable analysis (Chi^2^-test)

Variable	Categories	Total		Case flocks (n = 84)		Control flocks (n = 1414)		
		n	%	n	%	n	%	P-value
How often is concentrate offered?^b^	Twice daily	896	60.5	51	58.0	849	60.6	< 0.001
	Once daily	314	21.2	16	18.5	299	21.3	
	≥ Thrice daily	87	5.9	7	8.6	80	5.7	
	Automat	62	4.2	11	13.6	51	3.6	
	Not giving concentrate	123	8.3	2	1.2	122	8.7	
Ewes’ faecal consistency when lambing starts^a^	Firm pellets	1234	84.4	57	71.3	1177	85.1	0.001
	Soft paste or diarrhoea	229	15.7	23	28.8	206	14.9	
kg concentrate before lambing^a^	< 0.5 kg	726	49.0	29	34.9	697	49.8	0.004
	0.5–1 kg	640	43.2	41	49.4	599	42.8	
	> 1 kg	116	7.8	13	15.7	103	7.4	
Type of forage^a^	Silage and hay	326	21.8	9	10.7	317	22.5	0.008
	Hay	211	14.1	8	9.5	203	14.4	
	Silage	958	64.1	67	79.8	891	63.2	
Kg concentrate after lambing	< 1 kg	755	51.6	40	50.0	715	51.7	0.880
	1–1.5 kg	497	34.0	27	33.8	470	34.0	
	> 1.5 kg	210	14.4	13	16.3	197	14.3	

^a^Tested in multivariable model

^b^Intervening variable, not tested in the multivariable model

The attack rate of infectious arthritis among the case flocks was 5–10% in 69 flocks (82%), 11–20% in 12 flocks (14%), while three farmers (4%) reported that 21% or more of the lambs were affected. Lameness or swollen joints were reported as clinical signs in all the case flocks. In addition, “general apathy” was reported as a clinical sign in 25% of the flocks, and recumbency or reluctance to move was reported in 13% of the flocks. Only 6%, 3% and 1% reported navel infection, dyspnea and coughing, respectively.

**Fig. 1:**
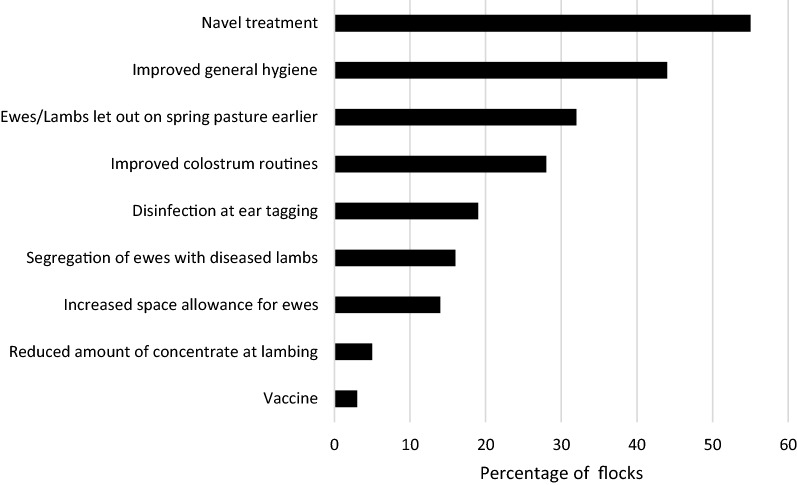
Preventive measures introduced in 195 of the 212 sheep flocks that had experienced an outbreak of infectious arthritis between 2015 and 2017. Sixty-six of these flocks were case flocks in 2018

Of the case flocks, 66 (79%) had experienced an outbreak of infectious arthritis in at least one of the lambing seasons before 2018 (Table 1). Unconditional logistic regression on the factor “previous outbreak” gave an OR of 29 (95% CI 16.5–50.6), but as this variable was considered an intervening factor it was not included in the multivariable model. Among the farmers that had an outbreak of arthritis in young lambs before 2018 (n = 212), 92% had introduced measures to prevent future outbreaks (Fig. 1). Disinfection of navels was the most commonly reported measure (55%).

In flocks with outbreaks in 2018 or one of the three previous seasons, the diagnosis of infectious arthritis was made by a veterinarian for 48% of the flocks, and by the farmer him-/herself for 46% of the flocks. Four percent had submitted samples for bacteriological culture from affected lambs, and 2% had submitted dead lambs for necropsy.

Antibiotic treatment was reported as administered to most of the affected lambs in 74 (89%) of the case flocks, and a few animals in seven (8%) case flocks. Respondents of 2 (2%) case flocks reported that no affected lambs had been treated with antibiotics. The most common route of administration of antibiotics was injection (n = 74, 88%), but affected lambs were treated *per os* in five case flocks (6%). The reported duration of treatment was 2–3 days (42%), 4–5 days (39%) or more than 5 days (16%).

In larger flocks, early ear tagging was more common. Among flocks with ˂ 200 lambs (n = 1012); 200–500 lambs (n = 426); and > 500 lambs (n = 57), 41%, 66% and 91% of the respondents, respectively, reported to perform ear tagging within 24 h after birth.

### Multivariable analysis

Altogether 44 variables were screened in the univariable analysis, and 20 were offered to the multivariable model (Tables 1, 2, 3, 4, and 5). Risk factors that remained in the final model are shown in Table 6. The overall likelihood ratio Chi^2^ test (5 df) P-value of the model was < 0.001.

**Table 6:** Final multivariable logistic regression model for flock risk factors for outbreaks of infectious arthritis in lambs

Variable	Categories	n cases	n controls	OR	95% CI
Flock size^a^				1.3	1.1–1.4
Flooring in lambing pen	Metal mesh flooring	23	525	Base	
	Plastic mesh flooring	38	235	3.0	1.7–5.3
	Other	16	418	0.9	0.5–1.7
Observed infections/pus around ear tag wounds	No (never/rarely)	39	874	Base	
	Yes (sometimes, often)	38	304	2.6	1.6–4.3
Lambing percentage	≤ 200	17	468	Base	
	> 200	60	710	2.0	1.1–3.5

^a^Number of ear-tagged lambs, divided by 100

None of the removed variables had a confounding effect on any variable parameter estimate in the final model. The interaction terms flock size * flooring and flock size * lambing percentage were non-significant (P > 0.05). The model showed acceptable fit according to the Hosmer–Lemeshow goodness of fit test, with chi squared (df 8) = 10.1 (P = 0.26). Examining the observations with Pearson residuals > 2 (n = 54), deviance > 2 (n = 43) or leverage > 3 * mean hat (n = 43) did not show any patterns with regard to values of explanatory variables. Refitting the model without different combinations of these observations had a small effect on OR estimates, and goodness of fit was still acceptable.

The geographical distribution of flocks corresponded well with the distribution of flocks in Norway (data not shown). Flock characteristics used to assess the representativeness of the respondents are presented in Table 7.

**Table 7:** Flock characteristics used to assess the representativeness of the respondents

Parameter	Respondents	Members of NSRSª
Flock size (winter-housed ewes), mean	79	86
Lamb mortality (%), mean	4.4	4.4
Breed composition (%)	74.4^b^	70^c^

^a^Norwegian Sheep Recording System

^b^Flocks with Norwegian White as main breed

^c^Percentage of ewes (members of the NSRS) that are Norwegian White

The median flock size (ear tagged lambs) was 138 in flocks with more than 40 lambs (n = 1498), and 141 in the dataset that was used in the multivariable analysis (n = 1178). The median overall lamb mortality was three percent in both groups.

## Discussion

This study confirms that outbreaks of infectious arthritis in lambs present an important animal health issue in Norway that needs to be managed to reduce the negative impacts on animal welfare, antibiotic usage, and profitability. Identification of flock-level risk factors is fundamental to development of evidence-based management strategies to prevent future outbreaks.

In this study, almost 6% of the included sheep flocks had experienced an outbreak of infectious arthritis in young lambs in 2018, fitting the characteristics of SDSD outbreaks. Assuming the respondents were representative for Norwegian sheep farmers with more than 40 lambs, and that 2018 was a representative year, the study suggests that 6% of Norwegian sheep flocks may be affected by an outbreak during the lambing season. This could be an overestimation of the true flock level prevalence, as the farmers that have experienced outbreaks are probably more likely to respond to the questionnaire. Without historical data it is not possible to evaluate whether this represents an increase compared to previous years or not.

Following pilot studies, the sheep health services suggested that outbreaks of arthritis are associated with large, intensively managed flocks [1]. This is supported in the present study, which confirms that flock size and a lambing percentage greater than 200 percent are risk factors for an outbreak. Farmers with larger flocks often have several hundreds of lambs born indoors within few weeks. A higher number of susceptible animals in a confined space will increase the risk of bacterial transmission. Another variable associated with intensive management; feeding of ewes with a high concentrate level prior to lambing, was the last factor to be eliminated from the model and was not statistically significant (LR-test P-value 0.08). Feeding with a high level of concentrate was not expected to predispose to outbreaks in itself, but through its association with ruminal acidosis and loose faecal consistency it was suspected it might negatively affect hygiene in the sheep shed and predispose to infections. Future studies involving farm visits can more accurately register and evaluate feeding routines, hygiene and faecal consistence than a questionnaire-based study.

Based on survey-data it was not possible to measure stocking density for each farm. Many respondents reported to have several sheds and to use provisional areas during lambing, leaving this variable difficult to assess. However, stocking density is expected to be relevant. Many veterinarians report to the sheep health services that over-crowding at lambing and a high turnover rate in the lambing areas are common features of flocks with outbreaks.

Not surprisingly, and in agreement with experiences of the sheep health services, flocks that have had outbreaks of infectious arthritis in previous years were at higher risk of outbreaks in the lambing season 2018. This could be associated with exposure to the same risk factors year after year, or the existence of a bacterial reservoir, presumably SDSD, in these flocks.

Anecdotal reports from some farmers and veterinary practitioners had indicated that plastic mesh flooring may be a risk factor for outbreaks. The current study confirms that flocks with lambing on plastic mesh flooring are at higher risk of arthritis outbreaks compared to flocks with lambing on other floor types. Compared to metal mesh flooring, which has been the most common floor in Norwegian sheep sheds, the plastic mesh floor has a larger surface area leaving more fluids, faeces and possibly bacterial biofilm on the surface in the lambing pen. Norwegian ewes often lamb directly onto the floor, without bedding material and are subsequently moved to individual pens with bedding. Possibly, the plastic floor contributes to transmission of SDSD to the lambs after birth.

Case reports have suggested early ear-tagging as a risk factor of infectious arthritis in lambs [12, 13]. While the univariable results in this study supported this, it was not verified in the multivariable analysis. Inflammatory reactions at the site of the ear tag, however, remained in the final model. Earlier research indicated that inflammatory reactions and infections around the ear tag are common findings following tagging of older lambs [22]. In the current study, no association was found between age at ear tagging and the occurrence of infections around the ear tag (Chi^2^ test, P = 0.58, not shown). However, 92% of the respondents reported that they tag their lambs within the first 5 days of life.

Seventy-nine percent of respondents with case flocks reported to have had an outbreak at least one of the previous seasons, and 92% of these had introduced preventive measures. This is probably the reason why some preventive measures recommended to prevent infectious arthritis in young lambs [3, 10], such as navel disinfection, ensuring adequate colostrum intake and disinfection of the skin at ear tagging, came out as risk factors rather than protective in the univariable results. Implementation of these measures are most likely a consequence, rather than a cause, of outbreaks.

Inadequate hand hygiene when performing lambing assistance was identified as a possible risk factor for outbreaks of infectious arthritis in in the univariable analysis but was not confirmed in the multivariable model. Rutherford et al. [15], suggested lambing assistance as a possible means of transmission of the bacteria within the flock, and proposed that vaginal colonization of the ewe may be an important reservoir. The proportion of ewes needing lambing assistance can be related to the feeding in late pregnancy and possibly genetic factors. In case flocks 53% of farmers claimed to assist more than 20% of their sheep in lambing while 40% of farmers in control flocks assisted more than 20% of their sheep. There was no association between inadequate hygiene and assisting a high percentage of ewes in lambing. The farmer’s attitude and level of experience probably play a role. Adherence to hygienic principles when providing lambing assistance may be an indicator of the general hygiene practice of the farmer, which could have wider implications for occurrence and transmission of infectious diseases. Hand washing and routines for changing gloves can also be related to the intensity of lambing, the availability of sufficient staff to handle many lambings in a short period of time, the barn design and the accessibility of washing facilities.

In the current study, the outcome of interest and the explanatory variables were collected using a questionnaire, and the sheep flocks were classified as cases or controls based on farmer reports. Only four and two percent of the case flock-respondents reported that they had submitted joint aspirates for culturing or lambs for necropsy, respectively. Without a bacteriological diagnosis from the flocks, we cannot rule out that some had outbreaks caused by other bacteria than SDSD. However, in light of results from the Norwegian pilot study, the age of the affected lambs (≤ 4 weeks) and the fact that outbreaks occurred during the lambing season while lambs were indoors, point to SDSD as the most likely cause.

The cut off for defining an outbreak was set at 5% of lambs affected. Clinical signs, age of diseased lambs and type of treatment were used to classify flocks correctly. The majority of farmers that reported outbreaks of infectious arthritis also described typical clinical signs. Only eight flocks were excluded because clinical signs were inconsistent with arthritis. There are likely to be differences in knowledge, routines for disease recording and accuracy of farmer recollections from the previous lambing season, and misclassifications of flocks may have occurred. In general, farmer-reported observations should be interpreted with some care, especially evaluation of their own management routines.

A questionnaire was used to reach out to as many farmers as possible, and because data on most of the explanatory variables were not available. Moreover, the outcome of interest, cases of infectious arthritis, is usually noted on mandatory paper-based health records only, and is not reported unless the animal receives veterinary treatment. In future, flock visits to observe housing and management, and to perform clinical examinations and sampling of affected animals for bacteriological culturing would be recommended.

Only farmers that were members of the Norwegian Sheep Recording System (NSRS) received the questionnaire, hence results may not be generalizable to non-member flocks. The representativeness of the NSRS data in comparison to the Norwegian sheep population has not been evaluated in detail, except on selected production parameters where differences were small [17]. Given that membership represents 40 percent of all sheep flocks in Norway, and knowledge generated from the long history of excellent census and mandatory health and medicines data for sheep and other livestock in Norway, the participating farms are considered representative of the management systems and flock sizes found across Norway.

There was no previous knowledge on the prevalence of infectious arthritis in lambs in Norwegian sheep flocks, and instead of carrying out a sample calculation and a subsequent sampling of herds, all members of the NSRS were invited to participate in the survey. Using the rule of thumb that a dataset with a rare outcome should contain at least 10 * (number of predictors in the model + 1) positive outcomes [21], our data with 77 case flocks would allow a model to be fitted with approximately 7 predictors, which is more than in the final model. Nevertheless, it is possible that additional management factors would have been included in the final model if the number of case flocks, and the statistical power, had been higher.

With the knowledge gained in this first study on risk factors for outbreaks of infectious arthritis in a Norwegian setting, further studies can be targeted towards the risk factors identified here as well as other interesting parameters that were not included in the final model.

## Conclusions

In this study, 5.6% of the sheep flocks had an outbreak of infectious arthritis in young lambs. Flocks that had suffered a previous outbreak were at higher risk of an outbreak in the lambing season 2018. The risk of outbreak increased with larger flock size, and in flocks with a lambing percentage greater than 200. In addition, lambing on plastic mesh flooring and infection or inflammation at the site of ear tags of lambs were associated with the risk of outbreak.

The risk factors identified in this study are characteristic of modern and intensively managed Norwegian sheep flocks. A distinguishing feature of Norwegian sheep farming is winter housing and indoor lambing.

An important task of future research will be to explore whether the risk factors identified in this study are connected to a possible reservoir of SDSD in sheep flocks. Investigations of bacterial sources on the animals and in the environment to enhance our knowledge of disease dynamics and bacterial transmission could pave the way for effective strategies for treatment, control and prevention of infectious arthritis in lambs.

## Supplementary information

**Additional file 1.** Questionnaire, translated version.

## Data Availability

The dataset is available from the corresponding author on reasonable request.
