# Supplementary material for: Flock-level risk factors for outbreaks of infectious arthritis in lambs, Norway 2018
Source: Acta Vet Scand. 2020 Nov 23;62:64. doi: 10.1186/s13028-020-00561-z (PMC7686670; doi:10.1186/s13028-020-00561-z)
Supplement: Supplementary file 1 — Additional file 1. Questionnaire, translated version. [file 13028_2020_561_MOESM1_ESM.docx]

| **Questionnaire – Flock level risk factors for outbreaks of infectious arthritis in lambs. Translated version** |
| --- |
| Date |
| 1.Farm id and postal code (if you want to answer anonymously, write only postal code) |
| 2.1.0: Number of winter-housed ewes 2016   \| 0-50 \| \| --- \| \| 51-100  101-200  201-400  >400 \| |
| 2.2.0: Number of winter-housed ewes 2017   \| 0-50 \| \| --- \| \| 51-100  101-200  201-400  >400 \| |
| 2.3.0: Number of winter-housed ewes 2018   \| 0-50 \| \| --- \| \| 51-100  101-200  201-400  >400 \| |
| 3. Sheep-breeds in the flock:   \| Norwegian White Sheep \| \| --- \| \| White short-tailed sheep  Other breeds \| |
| 4. Other animals on the farm:   \| Cattle \| \| --- \| \| Pigs  Poultry \| \| Horses \| \| Goats \| \| Llama, Alpaca \| \| Dog \| \| Cat \| \| Rabbit \| \| Other \| |
| 5. Housing type   \| Insulated \| \| --- \| \| Uninsulated  Polytunnels \| \| Open sided (3 walls) \| \| Combined \| \| Other \| |
| 6. Age of the shed   \| 10 years or newer \| \| --- \| \| Rebuild/modernized the last 10 years  Older than 10 years \| |
| 7. What is the size of the shed, in total area (m²) (don't include outside or additional areas used at lambing time) |
| 8. Provide approximate date for winter housing of ewes in autumn 2017 (dd.mm) |
| 9. Is the shed cleaned during the summer?   \| Annually \| \| --- \| \| Every second or third year  Every fourth to fifth year \| \| Less often than every fifth year \| |
| 10. Group size for the ewes – the last month before lambing, 2018   \| <10 \| \| --- \| \| 10-15  16-20 \| \| 21-30 \| \| >30 \| |
| 11. Number of lambs per ewe, 2018*   \| <1 \| \| --- \| \| 1-1,5  1,6-2.0 \| \| 2,1-2.5 \| \| >2.5 \|   *translated to: “Average lambing percentage in the flock, 2018” in the article   \| <100 \| \| --- \| \| 100-150  160-200 \| \| 210-250 \| \| >250 \| |
| 12. What type of forage was used in the housed period, 2018  Silage from round bales  Silage from pit  Hay  A mix of hay and silage |
| 13. Did you give concentrate to the ewes during the housed period, 2018?  Yes  No |
| 14.1.0. Amount of concentrate given daily to a ewe with two lambs: kg the week before expected lambing date, 2018   \| <0.5 kg \| \| --- \| \| 0.5-1 kg  1-1.5 kg \| \| 1.5-2 kg \| \| 2-2.5 kg  >2.5 kg \| |
| 14.2.0. Amount of concentrate given daily to a ewe with two lambs: kg the week after lambing, 2018   \| <0.5 kg \| \| --- \| \| 0.5-1 kg  1-1.5 kg \| \| 1.5-2 kg \| \| 2-2.5 kg  >2.5 kg \| |
| 15. How often is concentrate feed offered to ewes in last week before lambing, 2018  Once daily  Twice daily  Thrice daily  Automatic |
| 16. What was the faecal consistency of (most of the) ewes before lambing 2018?  Pellets  Pasty  Runny  I don’t know |
| 17. When did the lambing season start in 2018?   \| March \| \| --- \| \| April  May  June \| |
| 18. Approximate length of the lambing season 2018   \| 2-4 weeks \| \| --- \| \| 4-6 weeks  >6 weeks \| |
| 19.1.0 Number of lambs in the flock in 2018: Total number of lambs born in 2018 |
| 19.2.0 Number of lambs in the flock in 2018: Number of ear-tagged lambs |
| 19.3.0 Number of lambs in the flock in 2018: Number of lambs that died after ear tagging, but before 1 month of age |
| 20: Routines at lambing - statements:   - 20.1.0: As far as possible I leave ewe to revive lamb without any interference - 20.2.0: I remove mucus from nose and mouth of lamb with my hands - 20.3.0: I use straw/forage up nose to stimulate breathing (when necessary) - 20.4.0: I allow ewes and lambs to bond and monitor that they suck - 20.5.0: I routinely give colostrum to all lambs by bottle/tube first - 20.6.0: I give additional colostrum to weak lambs or those in high litter sizes only - 20.7.0: I always wash my hands and/or change gloves after handling diseased animals   Fully agree  Partly agree  Disagree |
| 21: Do you use stomach tubes?   \| No, never/rarely \| \| --- \| \| Yes occasionally  Yes, often \| |
| 22. Can you estimate what percentage of lambs you used stomach tube at least once in 2018?   \| 1-10 % \| \| --- \| \| 11-20 %  >20 %  I don’t know \| |
| 23. Routines for cleaning of bottle/teat/stomach tubes with soap and warm water:   \| Between every lamb \| \| --- \| \| Once daily  When needed \| |
| 24. Do you use colostrum supplement?  No, I don’t use colostrum supplement  Fresh/frozen sheep colostrum from another ewe  Commercial colostrum supplement(powdered product)  Fresh/frozen cow colostrum  Fresh/frozen goat colostrum  Other product (please state) |
| 25: Treatment of navels: Do you dip or spray the navels?  No, never/rarely  Yes, sometimes  Yes, always |
| 26: Type of navel treatment  Iodine based  Super7  Chlorhexidine  Spirit (alcohol)  Other, describe |
| 27: When do you usually spray/dip the navels?  At birth  Within 6 hours  After 6 hours |
| 28: Ear tagging: On average, how old are the lambs at ear tagging?  under 12 hours old  1 day  2 days  3-5 days  >5 days |
| 29: At ear tagging, do you disinfect the ear tags or the ear?  Yes  No |
| 30: Type of disinfectant used on ear/ ear tag:  Iodine based  Super7  Chlorhexidine  Spirit (alcohol)  Other, describe |
| 31: Have you observed infections around the ear tag (swollen, red and/or pus) in your flock?  No, never/rarely  Yes, on less than 10 % of the lambs  Yes, on 10-50 % of the lambs  Yes, on more than 50 % of the lambs |
| 32: Type of flooring in lambing pen  Plastic mesh flooring  Metal mesh flooring  Wooden slats  Straw bedded/deep litter  Other - please state |
| 33: Do you use lime/Stalosan on the floor in the shed/in lambing pens?  Yes, but only in the lambing period  Yes, throughout the housed season  No, never/rarely |
| 34: How well do these statements fit regarding the environment in the shed, in the lambing season of 2018:  34.1.0: The environment in the shed was as dry during lambing as it was before the lambing started  34.2.0: The environment in the shed was more humid during lambing compared to before  34.3.0: The environment in the shed was more humid and dirtier during lambing compared to before  34.4.0: There was higher humidity and temperature in the shed during lambing compared to before lambing.  Doesn’t fit at all  Fit for parts of the lambing period  Fit for the whole lambing period |
| 35: Type of bedding material in the lambing pens:  Not using bedding material  Straw  Saw dust  Hay  Other, describe |
| 36: Do you change the bedding materials in the lambing pens before the next lambing?  Always  Sometimes  Usually  Rarely/never |
| 37: Do you clean the lambing pens between lambings?  Never  Sometimes  Always |
| 38: Statements about the hygiene when performing lambing assistance  38.1.0: I wash my hands before performing lambing assistance  38.2.0: I use a new pair of disposable plastic gloves when performing lambing assistance  38.3.0: I routinely wash the vulva/perineum with water/soap  38.4.0: I wipe the vulva/perineum with wet-wipes/baby-wipe  38.5.0: I dry off the vulva/perineum with paper  Always  Sometimes  Never |
| 39: Can you estimate what percentage of lambings you needed to assist in 2018?  Less than 10 %  10-20 %  More than 20 % |
| 40: How long did ewes and lambs stay in lambing pens (days on average) in 2018?  1­-2 days  3-5 days  More than 5 days |
| 41: Before letting animals out onto pasture, what were lambs housed on:  Plastic mesh flooring  Metal mesh flooring   \| Wodden slats \| \| --- \| \| Straw bed \| \| Shavings deep litter \| \| Outdoors only \| \| Out in day, in at night \| \| Other, describe \| |
| 42: Did your flock have any cases of joint ill during the lambing 2018?  Yes  No |
| 43: Did your farm have any cases of joint ill in 2017 or earlier?  No  Yes, some cases every year  Yes, some cases, but not every year  Yes, we have had outbreaks (more than 5% of lambs affected) |
| 44: Which of the following years did you experience outbreak(s) of joint ill (more than 5% of lambs affected)  Before 2015  2015  2016  2017 |
| 45: Can you estimate the percentage of lambs affected:  45.1.0: In 2015  45.2.0: In 2016  45.3.0: In 2017  45.4.0: In 2018  < 5%  5-10 %  11-20 %  21-30 %  >30 % |
| 46: Have you introduced any measures to prevent outbreaks of joint ill?  No  Reduced amount of concentrate at lambing  Improved general hygiene at lambing  Improved colostrum routines  Treatment of navels  Vaccine  Increased space allowance for ewes  Disinfection of ear tag/ear when tagging  Ewes/lambs let out on spring pasture earlier  Segregated ewes with diseased lambs  Other, describe |
| 47: In connection to an outbreak of joint ill (more than 5 % of lambs affected), when did the first case occur?  Early in the lambing season  In the middle of the lambing season  Late in the lambing season |
| 48: How was joint ill diagnosed in your farm?  Recognised signs and diagnosed myself  Veterinary examination of live cases  Post-mortem by vet or sent for post-mortem  Sample collected from joint for bacterial culture |
| 49: What signs of joint ill did you see?  Lameness  Warm or swollen joint  Interdigital swelling  An inability to stand  Laboured breathing  Coughing  Navel infection  Scouring  General apathy  Other, describe |
| 50: At which age did you usually identify young lambs with signs of joint ill?  1-5 days old  6-10 days old  11-14 days old  15-20 days old  21 days or more |
| 51: Treatment of lambs with join ill:  The affected lambs were not treated  Only some of the affected lambs were treated  The majority of affected lambs were treated |
| 52: Length of treatment:  1 day  2-3 days  4-5 days  >5 days |
| 53: Type of treatment  Antibiotics – tablets  Antibiotics – injection  Combination of injection/tablets  Other |
| 54: Do you have other comments or information on joint ill in lambs? |
